# HMGB1 Acts in Synergy with Lipopolysaccharide in Activating Rheumatoid Synovial Fibroblasts via p38 MAPK and NF-****κ****B Signaling Pathways

**DOI:** 10.1155/2013/596716

**Published:** 2013-11-04

**Authors:** Zheng-Wen He, Yang-Hua Qin, Zhi-Wei Wang, Yan Chen, Qian Shen, Sheng-Ming Dai

**Affiliations:** ^1^Department of Laboratory Diagnosis, Changhai Hospital, Second Military Medical University, 168 Changhai Road, Shanghai 200433, China; ^2^Department of Rheumatology & Immunology, Changhai Hospital, Second Military Medical University, 168 Changhai Road, Shanghai 200433, China; ^3^Department of Orthopedics, Changhai Hospital, Second Military Medical University, Shanghai 200433, China

## Abstract

Synovial fibroblasts (SF) play a central role in the inflammatory and destructive process in rheumatoid arthritis (RA). High-mobility group box chromosomal protein 1 (HMGB1) or lipopolysaccharide (LPS) alone failed to induce significant changes in proliferation of cultured SF from RA patients, but premixed HMGB1 with LPS (HMGB1-LPS) significantly facilitated SF proliferation. HMGB1 alone failed to induce IL-6, MMP-3, and MMP-13 production in cultured SF but greatly enhanced LPS-induced expression of IL-6, MMP-3, and MMP-13 at both mRNA and protein levels. HMGB1-LPS synergistically upregulated TLR4 and receptor for advanced glycation endproducts (RAGE) expression on the surface of SF. Both blockers of TLR4 and RAGE significantly inhibited the synergistic effects of HMGB1-LPS on the production of IL-6 and MMPs, but blocking antibodies to TLR2 failed. HMGB1-LPS synergistically increased intracellular levels of phosphorylated p38 and phosphorylated I**κ**B. Furthermore, both NF-**κ**B inhibitor Bay11-7085 and p38 inhibitor SB203580 significantly suppressed the enhanced production of IL-6 and MMPs induced by HMGB1-LPS. In conclusion, HMGB1 acts in synergy with LPS to upregulate TLR4 and RAGE expression on the surface of SF in RA and then to augment IL-6, MMP-3, and MMP-13 production, which depends on p38 MAPK and NF-**κ**B activation.

## 1. Introduction

Rheumatoid arthritis (RA) is a systemic autoimmune disease characterized by chronic inflammation and cartilage and bone destruction in multiple joints. The abnormal proliferative synovial tissue serves as both the propagator of the immune response and the engine of tissue damage. Synovial fibroblasts (SF) in RA are one of the dominant cell types in the terminal layer of the hyperplastic rheumatoid synovium and at the sites of invasion into the adjacent cartilage and bone. RA synovial fibroblasts (RASF) actively contribute to inflammation, angiogenesis, and matrix degradation by producing proinflammatory cytokines, proangiogenic factors, and matrix degrading enzymes [1–3]. Proinflammatory factors produced by immune cells and RASF further induce the secretion of matrix-degrading enzymes and proinflammatory cytokines by RASF, contributing to joint erosion and enhancing the inflammatory cycle in RA. Potential mechanisms in RASF activation have been the focus of many studies, and some aspects have recently been uncovered. For instance, various proinflammatory factors, including tumor necrosis factor (TNF) *α*, interleukin (IL)-6, and IL-1*β*, as well as Toll-like receptor (TLR) ligands can activate RASF by inducing proinflammatory factors and matrix metalloproteinases (MMPs) [1–3]. However, knowledge about the crosstalk between the aforementioned factors underlying the activation of RASF in RA is very limited.

High-mobility group box 1 protein (HMGB1), a nonhistone nuclear protein, is a prototypic alarmin that is passively released from dying cells or actively secreted by activated macrophages and other myeloid cells. It is known that proinflammatory cytokines such as IL-1*β* or TNF*α* stimulate HMGB1 translocation into the cytoplasm and release in different cell types. Extracellular HMGB1 mediates inflammation via induction of cytokine and metalloproteinase production and recruitment and activation of inflammatory cells [4, 5].

Recent data show that HMGB1 can play a pivotal role in the pathogenesis of a wide variety of inflammatory conditions and may present a new target of therapy for RA and related rheumatic diseases [4–6]. The following observations support a pathogenic role for HMGB1 in RA: aberrant extranuclear HMGB1 expression occurs in the serum, synovial tissue, and synovial fluid of RA patients; aberrant synovial HMGB1 expression is downregulated by intra-articular corticosteroid injections; intraarticular injection of exogenous HMGB1 induces destructive arthritis in mice; HMGB1-targeted treatment attenuates arthritis in animal models and in particular ameliorates the structural damage [6–9]. However, the mechanisms underlying the pathologic effects of HMGB1 in RA are not fully elucidated. Moreover, it is still not fully elucidated how HMGB1 exerts its extracellular role. The issue is whether HMGB1 can mediate inflammation on its own, or whether it must be combined with other proinflammatory molecules to mediate inflammation. We and others found that pure HMGB1 failed to induce or minimally induce proinflammatory cytokine production in macrophages, but HMGB1 acts in synergy with IL-1*β* or endotoxin (a pathogen-associated molecule pattern), which binds to TLR4, to induce proinflammatory cytokine production in macrophages or SF [10–13]. Here, we studied whether there are any synergistic effects of HMGB1 and endotoxin (lipopolysaccharide, LPS) on the proliferation and biological function of RASF and tried to elucidate the underlying mechanisms responsible for the effects.

## 2. Materials and Methods

### 2.1. Reagents

Recombinant HMGB1 proteins were purchased from Sigma-Aldrich (St. Louis, MO, USA). We then detected the endotoxin contamination with *Limulus* amebocyte lysate (ZhanJiang A&C Biological, China), and only pure HMGB1, in which the endotoxin content must be <3 EU/mg, was used in the following experiments.

Fetal calf serum and Dulbecco's Modified Eagle's Medium (DMEM) were purchased from Invitrogen (Carlsbad, CA, USA). LPS from *Escherichia coli* serotype O55:B5, NF-*κ*B inhibitor Bay11-7085, Triton X-100, propidium iodide, and DNase-free RNase A were purchased from Sigma-Aldrich (St. Louis, MO, USA). SB203580, an inhibitor of p38 MAPK, was purchased from Calbiochem (Darmstadt, Germany). Blocking antibodies (Abs) against human TLR2, and TLR4, phycoerythrin (PE) conjugated Abs to TLR2, and allophycocyanin conjugated Abs to TLR4 were purchased from eBioscience (San Diego, CA, USA). Recombinant human RAGE Fc chimera (RAGE-Fc) was purchased from R&D Systems (Minneapolis, MN, USA). Allophycocyanin conjugated Abs to RAGE were purchased from Millipore (Billerica, MA, USA). 

### 2.2. Patients

Fresh synovial tissue specimens were obtained at the same time of knee joint replacement surgery from 10 patients with RA (mean age 56 years, range 36–70). All patients met the American College of Rheumatology 1987 revised criteria for the classification of RA [14]. All samples were obtained with informed consent from the patients, and the study was approved by the Ethics Committee of the Second Military Medical University.

### 2.3. Isolation and Culture of RASF

After careful removal of the adipose and fibrous tissue, synovial tissue fragments were minced and digested overnight on a horizontal shaker at 37°C in DMEM containing 1.0 mg/mL of bacterial collagenase. The cell suspensions were then filtered through a 70 *μ*m nylon filter and collected by centrifugation. The cells were washed and then resuspended with complete medium made of DMEM containing 10% fetal calf serum, 100 U/mL penicillin, and 100 *μ*g/mL streptomycin. The cells were counted, seeded into culture flasks (2.5 × 10^4^ cells/cm^2^), and cultured overnight in a humidified, 5% CO_2_ atmosphere at 37°C. The nonadherent cells were then washed out. Adherent cells were cultured in complete medium, and the culture medium was replaced every week. Upon confluence, cells were dispersed with trypsinization and then transferred to new plastic dishes in a split ratio of 1 : 3. For subsequent experiments, cells were used at passages 3–6, at which time they were a homogeneous population of fibroblasts. Cultured RASF were starved for 24 hours in DMEM supplemented with 1% fetal calf serum before experiments. RASF were incubated with 10 ng/mL of LPS and/or 100 ng/mL of HMGB1 for the indicated periods. When used in combination, LPS and HMGB1 were premixed for at least 30 min at 37°C before they were added into the culture medium. In some experiments, RASF were pretreated with blocking Abs or soluble receptors for 30 min or signal inhibitors for 1 h prior to the addition of LPS and/or HMGB1.

### 2.4. Flow Cytometric Analysis

Cells were harvested with a cell scraper and transferred to fluorescence-activated cell sorting tubes. For analysis of TLR2, TLR4, and RAGE expression on cell surface, RASF were then treated with Abs or isotype-matched control (2 *μ*g/mL) at 4°C for 35 minutes in the dark. After washing with PBS, samples were assayed using a FACSCalibur system (Becton Dickinson, Franklin Lakes, NJ, USA). Analysis was performed using CellQuest software (Becton Dickinson, Mountain View, CA, USA). 

For cell cycle phase analysis, RASF were detached with trypsin treatment and fixed with 70% ice-cold ethanol. The cells were digested with 100 *μ*g/mL RNase at 37°C for 20 minutes and then stained with 12.5 *μ*g/mL propidium iodide for 30 minutes in the dark. Filtered samples were then analyzed for cycle content on a FACSCalibur cell sorter, using CellQuest software, and the percentages of cells in the G1, S, and G2/M phases were determined using ModFit LT software (Verity Software House, Topsham, ME, USA).

### 2.5. Cell Proliferation Assay

Cell proliferation was measured as previously described, using Cell Counting Kit-8 (CCK-8) according to the instructions of the manufacturer (Dojindo Laboratories, Kumamoto, Japan) [15]. 

### 2.6. Quantitative PCR

Total RNA was purified with TRIzol reagent (Invitrogen Life Technologies, Carlsbad, CA, USA) and cDNA was synthesized using PrimeScript reverse transcription reagents (TaKaRa, Dalian, China). Real-time PCR was performed on a Rotor-Gene 6000 Real-Time PCR using SYBR Premix EX Taq reagent (TaKaRa, Dalian, China). Primer sequences of MMP-3, MMP-13, IL-6, and *β*-acting were listed in Table 1. The housekeeping gene *β*-actin was used to normalize all tested genes, and datum quantification was performed using the ^ΔΔ^CT method.

### 2.7. ELISA

The levels of total MMP-3, MMP-13, and IL-6 released from RASF into the culture supernatants were evaluated by ELISA with commercial kits according to the instructions of the manufacturers. The ELISA kits for MMP-3 were purchased from R&D System (Minneapolis, MN, USA) and for MMP-13 and IL-6 were from eBioscience (San Diego, CA, USA). The sensitivities of the kits were 9 pg/mL for MMP-3, 0.5 ng/mL for MMP-13, and 0.5 ng/mL for IL-6. 

### 2.8. Western Blotting

RASF were lysed in a buffer containing 1.0% (vol/vol) Nonidet-P40, 150 mM NaCl, 20 mM Tris-HCl (pH 7.5), 1 mM EDTA, and a protease inhibitor mixture (Roche, Basel, Switzerland). Proteins (25 *μ*g) in cell lysates were separated by SDS-PAGE and transferred to polyvinylidene difluoride membranes. Membranes were blotted with the appropriate Abs and were visualized with an ECL Western Blotting System (Pierce Protein Research Products, Rockford, IL, USA).

### 2.9. Statistical Analysis

Data are expressed as mean ± SD. All experiments were repeated independently at least three times. Statistical comparisons were made using unpaired Student's *t*-test for two groups or one-way ANOVA followed by Student-Newman-Keuls' multiple test or Dunnett's test for multigroups. Two-tailed *P* < 0.05 was considered statistically significant. The software program GraphPad Prism version 5 for Windows (GraphPad Software, San Diego, CA, USA) was used for all tests.

## 3. Results

### 3.1. HMGB1 Acted in Synergy with LPS to Stimulate Proliferation of RASF

**Figure 1:**
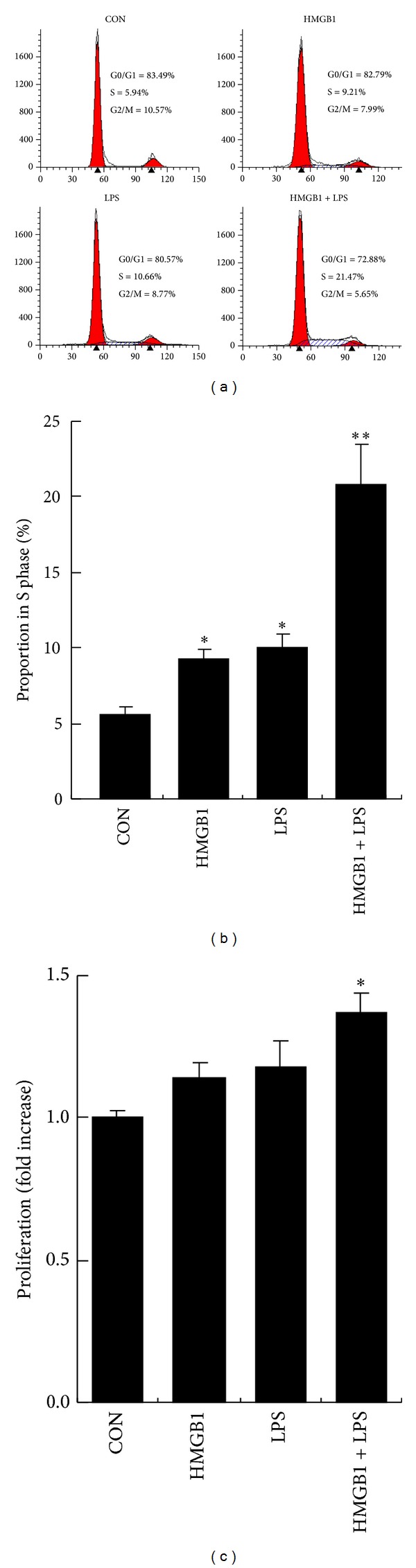
HMGB1 acted in synergy with LPS to stimulate the proliferation of SF. CON: control; HMGB1: high-mobility group box 1 protein; LPS: lipopolysaccharide. Cultured synovial fibroblasts (SF) were isolated from synovium obtained from patients with rheumatoid arthritis (RA): and cultured in vitro for 3–6 passages. SF were incubated with 10 ng/mL of LPS and/or 100 ng/mL of HMGB1 for 24 h. (a) and (b) SF were stained with propidium iodide for flow cytometric analysis. The percentages of cells in the G1, S, and G2/M phases of the cell cycle were determined using ModFit LT software. Representative histograms (a) and the percentages of the cells in S phase (b) were shown. (c) Cell proliferation was analyzed using commercially available Cell Counting Kit-8. Data are expressed as mean ± SD (*n* = 3). *P < 0.05 and **P < 0.01 compared with control (unstimulated SF). Results shown are representative of 4 independent experiments.

When the cultured RASF were stimulated with LPS (10 ng/mL) or HMGB1 (100 ng/mL) alone for 24 h, cell cycle analysis showed that the proportion of the cells in S phase significantly increased (Figures 1(a) and 1(b)), but no significant changes in cell proliferation rates were found (Figure 1(c)). HMGB1 (100 ng/mL) in combination with LPS (10 ng/mL) (HMGB1-LPS) further increased the proportion of the cells in S phase and significantly increased the proliferation rate of RASF (Figures 1(a)–1(c)). 

### 3.2. HMGB1 Acted in Synergy with LPS to Induce Production of IL-6 and MMPs

**Figure 2:**
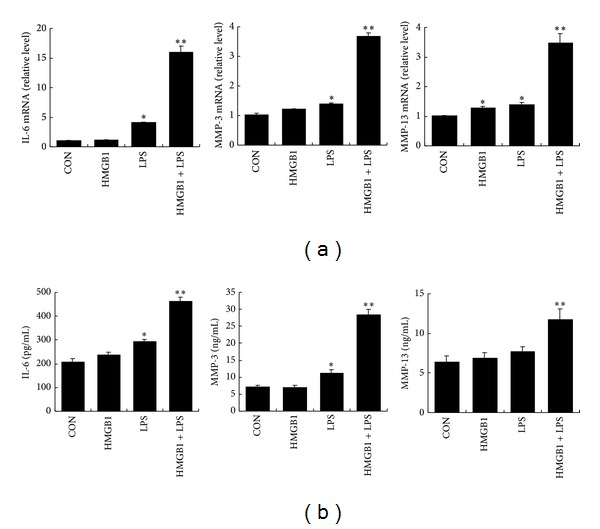
HMGB1 acted in synergy with LPS to induce IL-6, MMP-3, and MMP-13 expression in cultured SF. IL-6: interleukin-6; MMP: matrix metalloproteinase. (a) mRNA expression levels of IL-6, MMP3, and MMP-13 were analyzed by quantitative real-time RT-PCR. Total RNA was isolated from cultured SF stimulated with 10 ng/mL of LPS and/or 100 ng/mL of HMGB1 for 3 h. (b) The concentrations of IL-6, MMP-3, and MMP-13 proteins in culture supernatants were measured by ELISA using commercial kits. The supernatants were collected after the cultured SF were stimulated with 10 ng/mL of LPS and/or 100 ng/mL of HMGB1 for 48 h. Data are expressed as mean ± SD (*n* = 4). *P < 0.05 and **P < 0.01 compared with control (unstimulated SF).

After 3 h treatment, LPS (10 ng/mL) alone significantly increased IL-6 mRNA, MMP-3 mRNA and MMP-13 mRNA expression levels, and HMGB1 (100 ng/mL) alone significantly increased MMP-13 mRNA expression level. However, no significant effect of HMGB1 on IL-6 mRNA, and MMP-3 mRNA expression was found. HMGB1 greatly augmented the enhancing effects of LPS on mRNA expression levels of IL-6, MMP-3, and MMP-13 (Figure 2(a)). After 48 h treatment, LPS (10 ng/mL) alone also significantly increased the protein levels of IL-6, MMP-3, and MMP-13 in culture supernatants, while HMGB1 (100 ng/mL) alone failed to increase the protein levels of them. Significantly synergistic effects of HMGB1-LPS on the secretion of IL-6, MMP-3, and MMP-13 proteins from RASF were found (Figure 2(b)).

### 3.3. HMGB1 Acted in Synergy with LPS to Upregulate RAGE and TLR4 Expression

**Figure 3:**
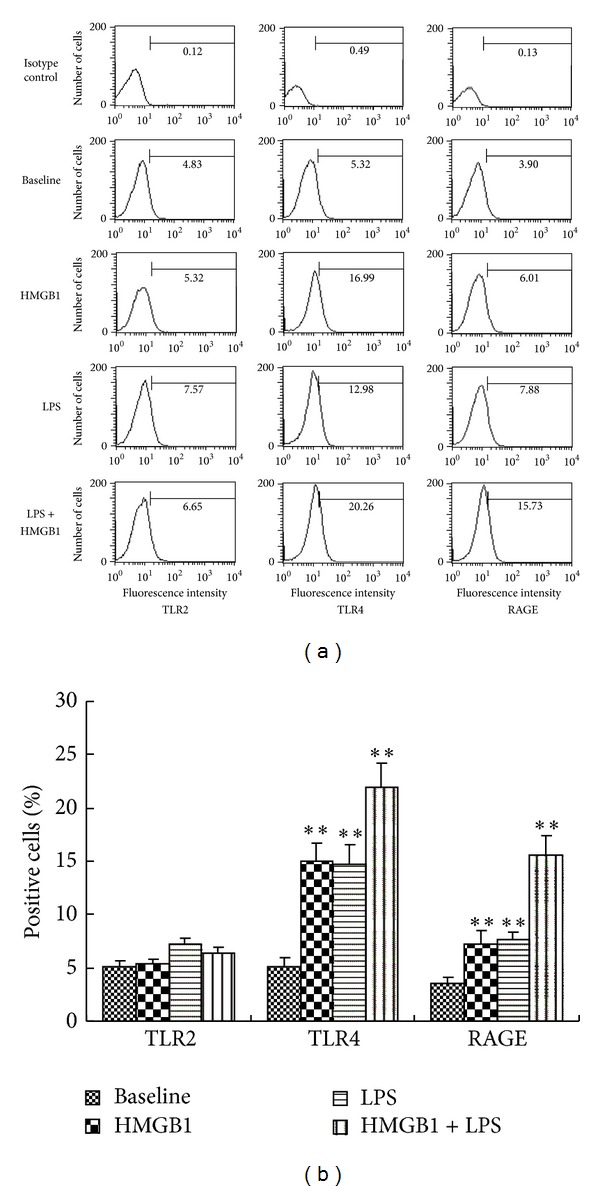
HMGB1 and LPS upregulated TLR2, TLR4, and RAGE expression on the surface of cultured SF. TLR: Toll-like receptor; RAGE: receptor for advanced glycation endproducts. SF were stimulated with 10 ng/mL of LPS and/or 100 ng/mL of HMGB1 for 24 h. The expression levels of molecules on the surface of SF were analyzed by flow cytometric analysis using specific antibodies. Representative histograms (a) and the percentages of the positive cells (b) were shown. Data are expressed as mean ± SD (*n* = 4). *P < 0.05 and **P < 0.01 compared with control (unstimulated SF).

The biological effects of LPS are mediated by TLR4, and those of HMGB1 may be mediated by RAGE, as well as TLR2 and TLR4 [16, 17]. To further elucidate the underlying mechanisms for the synergistic effects between HMGB1 and LPS, we studied whether the protein expression levels of their receptors are regulated by each other. Flow cytometric analysis showed that LPS significantly upregulated RAGE, TLR2, and TLR4 expression on the surface of RASF. HMGB1 significantly upregulated TLR4 and RAGE expression but failed to affect TLR2 expression. When RASF were treated with HMGB1-LPS, significantly synergistic upregulation of TLR4 and RAGE expression, especially of TLR4 expression, was found (Figure 3). No significantly synergistic upregulation of TLR2 was induced by HMGB1-LPS.

### 3.4. Blockers of RAGE and TLR4 Inhibited the Synergistic Effects of HMGB1-LPS

**Figure 4:**
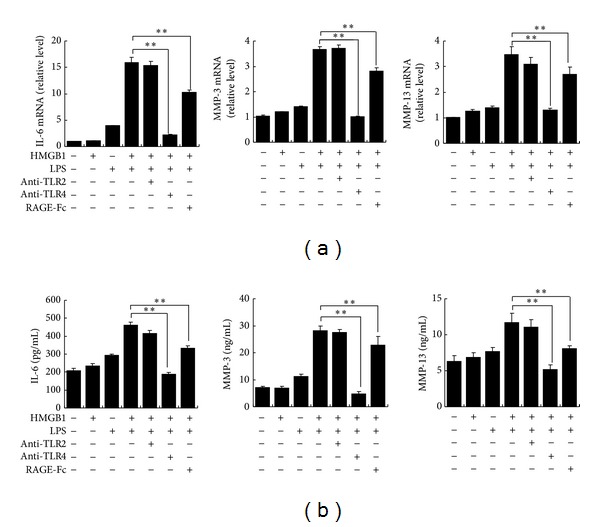
Synergistic effects of HMGB1-LPS were inhibited by blockers of TLR4 and RAGE. Cultured SF were pretreated with anti-TLR2 (10 *μ*g/mL), anti-TLR4 (10 *μ*g/mL), or RAGE-Fc (10 *μ*g/mL) for 30 min and then incubated with 10 ng/mL of LPS and/or 100 ng/mL of HMGB1 for 3 h (a) or 48 h (b). (a) mRNA expression levels of IL-6, MMP3, and MMP-13 were analyzed by quantitative real-time RT-PCR. (b) The concentrations of IL-6, MMP-3, and MMP-13 proteins in culture supernatants were measured by ELISA. Data are expressed as mean ± SD (*n* = 4). **P < 0.01 compared with SF stimulated with HMGB1 plus LPS.

To further assess which receptor was involved in the synergistic response, we applied blocking Abs (anti-TLR2 and anti-TLR4) or soluble receptor (RAGE-Fc) to interrupt the binding of TLR2, TLR4, or RAGE on the surface of RASF to their ligands. As a result, pretreatment with anti-TLR4 Abs almost completely abolished the production of IL-6 and MMPs from RASF induced by HMGB1-LPS, whereas pretreatment with anti-TLR2 Abs did not affect the production. Pretreatment with RAGE-Fc also significantly inhibited the production of IL-6 and MMPs from RASF induced by HMGB1-LPS (Figure 4). 

### 3.5. Activation of p38 MAPK and NF-*κ*B Mediated the Synergistic Effects of HMGB1-LPS

**Figure 5:**
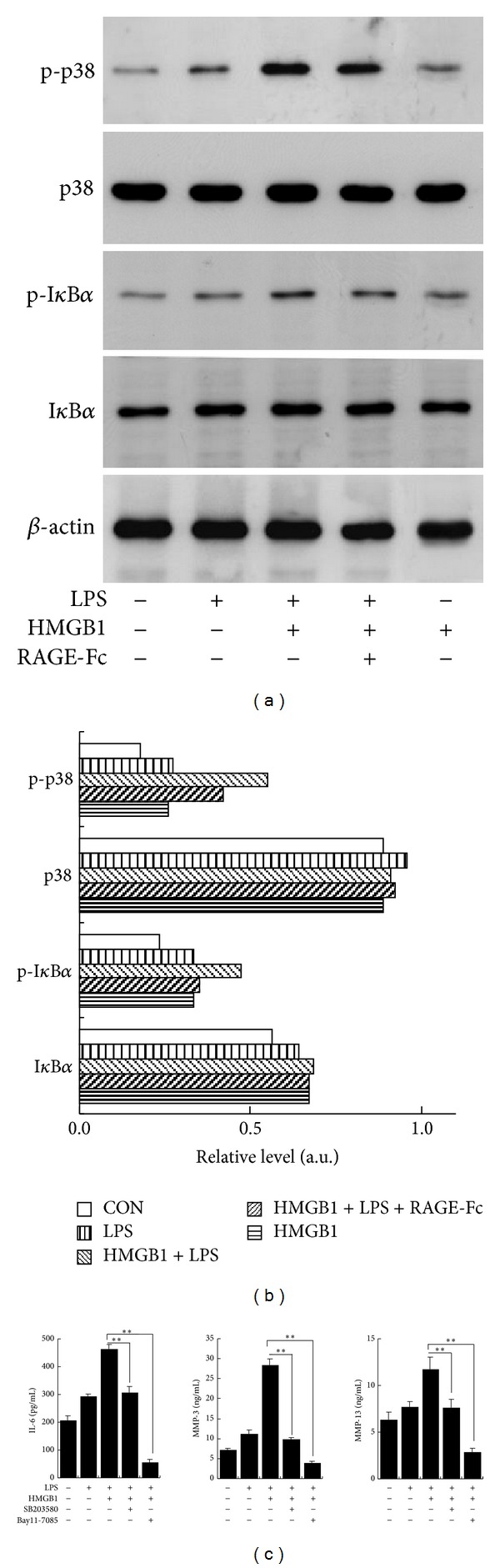
HMGB1 and LPS synergistically activated NF-*κ*B and p38 MAPK. MAPK: mitogen-activated protein kinase; p-p38: phosphorylated p38; p-I*κ*B: phosphorylated I*κ*B. (a) Levels of intracellular molecules in whole cell lysates were analyzed by Western blotting after the SF were stimulated with 10 ng/mL of LPS and/or 100 ng/mL of HMGB1 for 30 min. To test whether RAGE mediate the effect of HMGB1 plus LPS, SF were pretreated with RAGE-Fc (10 *μ*g/mL) for 30 min prior to the stimulation with HMGB1 plus LPS. (b) Densitometry analysis of the Western blot bands which was adjusted by the density of corresponding band of *β*-actin. (c) Cultured SF were pretreated with NF-*κ*B inhibitor Bay11-7085 (20 *μ*M) or p38 inhibitor SB203580 (10 *μ*M) for 1 h and then incubated with 10 ng/mL of LPS and/or 100 ng/mL of HMGB1 for 48 h. The concentrations of IL-6, MMP-3, and MMP-13 proteins in culture supernatants were measured by ELISA. Data are expressed as mean ± SD (*n* = 4). *P < 0.05 and **P < 0.01 compared with SF stimulated with HMGB1 plus LPS.

To study whether p38 MAPK and NF-*κ*B activation is involved in intracellular signal transduction of HMGB1-LPS, we analyzed expression levels of p38 MAPK, I*κ*B*α*, and their phosphorylated forms by Western blotting. The phosphorylated levels of p38 MAPK and I*κ*B*α* in RASF treated with HMGB1-LPS were higher than those of RASF treated with LPS alone or HMGB1 alone. Pretreatment with RAGE-Fc significantly inhibited the phosphorylation of p38 MAPK and I*κ*B*α* induced by HMGB1-LPS (Figures 5(a) and 5(b)). Both pretreatments with NF-*κ*B inhibitor Bay11-7085 (20 *μ*M) and p38 MAPK inhibitor SB203580 (10 *μ*M) significantly suppressed the enhanced production of IL-6, MMP-3, and MMP-13 from RASF induced by HMGB1-LPS (Figure 5(c)).

## 4. Discussion

HMGB1 was cloned initially in 1991 by Merenmies et al. [18] and has been revealed to be a nuclear DNA-binding protein, participating in stabilization of the nucleosome structure, regulation of gene transcription [19], and modulation of steroid hormone receptor activity [20, 21]. Recently, HMGB1 has been identified as a potent proinflammatory cytokine that controls the activation and chemotaxis of inflammatory cells and stimulates the synthesis of proinflammatory cytokines. Nevertheless, recent studies suggest that HMGB1 alone demonstrates minor proinflammatory activity, which is potentiated through binding to IL-1*β*, TLR4 ligand LPS, TLR9 ligand CpG-DNA, and other inflammatory mediators [10–12, 22]. This notion was further supported by the present study and other reports. Wähämaa et al. [13] reported that high concentration of HMGB1 (4 *μ*g/mL) failed to induce TNF production in cultured SF, whereas HMGB1 (4 *μ*g/mL) significantly augmented TNF production induced by LPS (1–100 ng/mL). Our data demonstrated that there were no significant effects of HMGB1 on cell proliferation or the production of IL-6, MMP-3, and MMP-13 in cultured RASF, but there were strong enhancing effects of HMGB1 on cell proliferation and the production of IL-6 and MMPs in RASF induced by TLR4 ligand LPS. When we added HMGB1 and LPS simultaneously into culture medium, no significantly synergistic effects between them were found (data not shown). Moreover, we and others have demonstrated that formation of HMGB1-LPS complex is essential for their synergistic effects in macrophages in previous reports [11, 12]. So LPS and HMGB1 were premixed before HMGB1-LPS was used in the present study. The amount of HMGB1 (100 ng/mL) used in our study corresponds to the level record in RA synovial fluid, ranging from 10 to 300 ng/mL [23].

Recent data show that HMGB1-mediated functions are conveyed via multiple receptors such as RAGE and several members of the TLR family including TLR2 and TLR4 [6, 24–26]. To explore the mechanism underlying the synergistic effects of HMGB1-LPS complex, we analyzed whether the expression levels of RAGE, TLR2, and TLR4 on the surface of RASF were modulated by HMGB1-LPS. As a result, synergistic effects of HMGB1-LPS on upregulation of RAGE and TLR4 expression were found. In particular, TLR4 expression was upregulated to the greatest extent. The synergistic effects of HMGB1-LPS on IL-6 and MMPs production were greatly inhibited by blocking Abs to TLR4, partially inhibited by soluble receptor RAGE-Fc but not inhibited by blocking Abs to TLR2. No inhibitory effect of TLR2 blocker correlated with upregulation of TLR2 by HMGB1-LPS in the present study. These data suggest that TLR4 and RAGE rather than TLR2 mediate the effects of HMGB1-LPS. However, it cannot be determined whether HMGB1 binds to TLR4 in RASF, because blocking Abs to TLR4 can inhibit the synergistic effects of HMGB1-LPS by interrupting the binding of LPS to TLR4. In previous reports, TLR4 may be activated by minimal amount of endotoxin in the preparations of HMGB1 rather than HMGB1 itself. We have demonstrated that TLR4 is not involved in the synergistic effects of HMGB1-IL-1*β* complex in macrophages [12]. Taken together, HMGB1-LPS upregulated TLR4 and RAGE expression, which may be responsible for the synergistic effects on the enhanced production of IL-6 and MMPs in RASF.

To further determine the possible mechanism of action of HMGB1-LPS, we explored intracellular signal transduction pathways. In the presence of IL-1*β*, HMGB1 enhanced the phosphorylated p38 MAPK level and significantly potentiated NF-*κ*B activation in SF from patients with osteoarthritis [27]. In particular, transcription of IL-6 and MMPs is NF-*κ*B and p38 MAPK dependent [28–30]. So we focused on NF-*κ*B and p38 activation. Classical NF-*κ*B activation involves phosphorylation of I*κ*B*α*, ubiquitination and subsequent degradation of phosphorylated-I*κ*B*α* from NF-*κ*B subunits, and translocation of NF-*κ*B subunits to the nucleus. We found that HMGB1-LPS synergistically enhanced the levels of phosphorylated p38 and phosphorylated I*κ*B*α*, suggesting enhanced activation of p38 and NF-*κ*B. Both NF-*κ*B inhibitor and p38 inhibitor significantly suppressed the enhanced production of IL-6 and MMPs induced by HMGB1-LPS in RASF, further supporting that HMGB1-LPS activates NF-*κ*B and p38. However, the present study does not exclude other possible signaling pathways involved in the effects of HMGB1-LPS. For example, intracellular ERK1/2 is involved in the production of IL-6 and MMPs from RASF induced by IL-1*β* [31], but we did not check the changes in expression level of phosphorylated ERK1/2.

Previous studies have demonstrated that proinflammatory cytokines such as IL-1*β* or TNF*α*, as well as LPS and oxidative stress, which are also involved in the pathogenesis of RA, stimulate HMGB1 translocation into the cytoplasm and release in different cell types, such as macrophages, dendritic cells, synoviocytes, and chondrocytes in arthritic joints [32–34]. Furthermore, there is abundant evidence that blockade of extracellular HMGB1 suppresses disease progression in experimental arthritis [8, 9, 35]. Except that HMGB1 induces proinflammatory cytokines and chemokines, RAGE activation by HMGB1 results in increased invasiveness of SF from RA patients [36]. Here, we demonstrated that HMGB1-LPS stimulates RASF proliferation and enhances the production of proinflammatory cytokines and MMPs from RASF, which puts new insight into the mechanisms of HMGB1 in the pathogenesis of RA. However, the physiopathological roles of HMGB1 in the RA have not been well elucidated yet. 

In conclusion, HMGB1 acts in synergy with LPS to induce p38 MAPK and NF-*κ*B activation and to upregulate RAGE and TLR4 expression in RASF. These effects result in enhanced production of proinflammatory cytokines and catabolic enzymes that would contribute to synovitis and articular destruction during RA process.

## Figures and Tables

**Table 1:** Sequences of PCR primers.

Gene	Primer (5′-3′)
MMP-3	fw: GGGTGAGGACACCAGCATGA rv: CAGAGTGTCGGAGTCCAGCTTC
MMP-13	fw: TTGATGATGATGAAACCTGGACAAG rv: TTGCCGGTGTAGGTGTAGATAGGAA
IL-6	fw: AAGCCAGAGCTGTGCAGATGAGTA rv: TGTCCTGCAGCCACTGGTTC
*β*-actin	fw: TGGCACCCAGCACAATGAA rv: CTAAGTCATAGTCCGCCTAGAAGCA

PCR: polymerase chain reaction; fw: forward; rv: reverse; IL: interleukin; MMP: matrix metalloproteinase.

## Abbreviations

Abs: Antibodies
ELISA: Enzyme-linked immunosorbent assay
HMGB1: High-mobility group box protein 1
IL: Interleukin
LPS: Lipopolysaccharide
MAPK: Mitogen-activated protein kinase
MMPs: Matrix metalloproteinases
PCR: Polymerase chain reaction
p-I*κ*B: Phosphorylated I*κ*B
p-p38: Phosphorylated p38 MAPK
RA: Rheumatoid arthritis
RAGE: Receptor for advanced glycation endproducts
SF: Synovial fibroblasts
TLR: Toll-like receptor
TNF: Tumour necrosis factor.
